# TMEFF2 shedding is regulated by oxidative stress and mediated by ADAMs and transmembrane serine proteases implicated in prostate cancer

**DOI:** 10.1002/cbin.10832

**Published:** 2017-08-21

**Authors:** Katarzyna Gaweł‐Bęben, Nazim Ali, Vincent Ellis, Gloria Velasco, Zaruhi Poghosyan, Ann Ager, Vera Knäuper

**Affiliations:** ^1^ School of Medicine University of Information Technology and Management in Rzeszow 2 Sucharskiego Str. 35‐225 Rzeszow Poland; ^2^ School of Dentistry College of Biomedical and Life Sciences Cardiff University Cardiff CF14 4XY United Kingdom; ^3^ School of Medicine University of Keele Keele ST5 5BG United Kingdom; ^4^ School of Biological Sciences University of East Anglia Norwich Research Park Norwich NR4 7TJ United Kingdom; ^5^ Departamento de Bioquímica y Biología Molecular Facultad de Medicina Universidad de Oviedo 33006 Oviedo Spain; ^6^ School of Medicine College of Biomedical and Life Sciences Cardiff University Cardiff CF14 4XY United Kingdom

**Keywords:** ADAM, hepsin, matriptase‐1, oxidative stress, TMEFF2

## Abstract

TMEFF2 is a type I transmembrane protein with two follistatin (FS) and one EGF‐like domain over‐expressed in prostate cancer; however its biological role in prostate cancer development and progression remains unclear, which may, at least in part, be explained by its proteolytic processing. The extracellular part of TMEFF2 (TMEFF2‐ECD) is cleaved by ADAM17 and the membrane‐retained fragment is further processed by the gamma‐secretase complex. TMEFF2 shedding is increased with cell crowding, a condition associated with the tumour microenvironment, which was mediated by oxidative stress signalling, requiring jun‐kinase (JNK) activation. Moreover, we have identified that TMEFF2 is also a novel substrate for other proteases implicated in prostate cancer, including two ADAMs (ADAM9 and ADAM12) and the type II transmembrane serine proteinases (TTSPs) matriptase‐1 and hepsin. Whereas cleavage by ADAM9 and ADAM12 generates previously identified TMEFF2‐ECD, proteolytic processing by matriptase‐1 and hepsin produced TMEFF2 fragments, composed of TMEFF2‐ECD or FS and/or EGF‐like domains as well as novel membrane retained fragments. Differential TMEFF2 processing from a single transmembrane protein may be a general mechanism to modulate transmembrane protein levels and domains, dependent on the repertoire of ADAMs or TTSPs expressed by the target cell.

**Abbreviations**
ADAM: a disintegrin and metalloproteinase
TMEFF2: transmembrane protein with EGF‐like and two follistatin‐like domains 2
TTSP: type II transmembrane serine protease

## Introduction

TMEFF2, a type I transmembrane protein with two follistatin and epidermal growth factor (EGF) domains, is expressed selectively in the adult brain and prostate (Horie et al., 2000; Liang et al., 2000), with elevated TMEFF2 expression in prostate cancer (PCa) cell lines and clinical samples (Glynne‐Jones et al., 2001; Gery et al., 2002; Afar et al., 2004). However, the role of TMEFF2 in PCa development and progression remains unclear, and TMEFF2 activity may depend on disease stage and/or post‐transcriptional regulation. The ectodomain of TMEFF2 (TMEFF2‐ECD), comprised of the two follistatin and EGF domains, is cleaved from the cell surface by ADAM17 and the membrane‐retained fragment undergoes further processing by the gamma‐secretase complex (Ali and Knäuper, 2007). Shedding of TMEFF2‐ECD is induced by pro‐inflammatory cytokines TNFα and IL‐1β (Lin et al., 2003) or phorbol esters (Ali and Knäuper, 2007), which are known to upregulate ADAM‐mediated protein shedding (Brose and Rosenmund, 2002).

Ectodomain shedding may, at least partially, be responsible for pro‐ or anti‐proliferative TMEFF2 functions in PCa. Overexpression of full length, transmembrane TMEFF2 in PCa cells impairs proliferation due to an interaction between the cytoplasmic domain of TMEFF2 and sarcosine dehydrogenase (SARDH). This interaction results in decreased levels of sarcosine (Chen et al., 2011a; Green et al., 2013), an amino acid associated with PCa progression (Sreekumar et al., 2009). Full length TMEFF2 also attenuates the migratory properties of PCa cells (Chen et al., 2014), indicating a tumour suppressor function. However, the TMEFF2‐ECD released, due to shedding, may act as a soluble growth factor. Indeed, treatment of HEK293 cells with purified recombinant TMEFF2‐ECD stimulated ERK activation and increased their proliferation rate (Ali and Knäuper, 2007; Chen et al., 2011a). On the other hand, conditioned medium of cells expressing TMEFF2‐ECD reduced p‐ERK levels in RWPE1 cells in response to PDGF‐AA treatment (Chen and Ruiz‐Echevarría, 2013) which was also reported to suppress PDGF‐AA stimulated growth of NR6 fibroblasts (Lin et al., 2011). While this points to TMEFF2 possessing opposing biological roles the molecular mechanism underlying this dual functionality is unclear. We hypothesise that TMEFF2's biological functions may be regulated by differential proteolysis, which generates not only TMEFF2‐ECD but additional protein fragments, which may modulate mitogenic signalling. Indeed, additional soluble TMEFF2 forms can arise from alternative splicing, generating a soluble protein composed of the FS‐I module and a truncated FS‐II module (Quayle and Sadar, 2006).

To address the hypothesis that TMEFF2 may undergo differential proteolysis we focused our investigation on proteases contributing to the pathogenesis of PCa. These include members of the disintegrin and metalloproteinase family, ADAM9 (Peduto et al., 2005; Fritzsche et al., 2008), ADAM12 (Peduto et al., 2006) and ADAM15 (Lucas and Day, 2009) as well as membrane associated serine proteases implicated in PCa, such as type II transmembrane serine proteases (TTSPs) (Webb et al., 2011) or the GPI‐anchored prostasin (Chen et al., 2004). Prominent TTSPs involved in PCa progression include matriptase‐1, matriptase‐2 and hepsin. Matriptase‐1 overexpression correlates with Gleason score (Riddick et al., 2005) promoting cell invasion, metastasis and prostate tumour growth (Sanders et al., 2006; Ko et al., 2015) by regulating MET signalling in PCa. Interestingly, matriptase‐1 interacts with a close relative of TMEFF2, TMEFF1, where the EGF‐like domain of TMEFF1 binds to the matriptase‐1 CUB domain (Ge et al., 2006). Matriptase‐2 also contains CUB domains and is implicated in PCa cell behaviour (Sanders et al., 2010). Significant overexpression of hepsin is common in 90% of PCa tumours, correlating with Gleason score, serum PSA levels as well as early relapse following radical prostatectomy (Dhanasekaran et al., 2001; Goel et al., 2011). In contrast, prostasin levels are high in normal prostate epithelial cells and decrease in PCa (Takahashi et al., 2003). We therefore tested the hypothesis that TMEFF2 is cleaved at different sites by ADAMs and TTSPs and we provide evidence of complex TMEFF2 proteolysis by these proteases that may impact the biological function of TMEFF2 reported in the literature.

## Materials and methods

### Reagents

PMA and the NADPH oxidase inhibitor apocynin (APOC) were from Sigma–Aldrich. N‐acetylcysteine (NAC), p38 inhibitor (SB203580) and JNK inhibitor (SP600125) and the broad spectrum metalloproteinase inhibitor GM6001 were from Calbiochem. ADAM10 and ADAM17 inhibitors GI254023X and GW280264X were a gift from Dr. Augustin Amour and GlaxoSmithKline. DMEM and Ham's F12 cell media were from Lonza, FBS and hygromycin B from Invitrogen. FuGENE 6 Transfection Reagent was from Roche.

### Expression constructs, cell culture, transient transfection and Western blotting

ADAM9 and ADAM12 expression constructs were a kind gift from Dr. Carl Blobel. The cloning of ADAM15 A, B, C isoforms and ADAM15 B E/A inactive mutant into pcDNA4‐V5/His vector was described previously (Zhong et al., 2008; Maretzky et al., 2009). The corresponding coding sequences were sub‐cloned into pcDNA5/FRT/Flag‐His plasmid using *HindIII* and *XhoI*. Matriptase‐2 expression plasmid was described in Folgueras et al. (2008). Matriptase‐1, hepsin and prostasin plasmids were described in Gray et al. (2014) and used to generate constructs expressing inactive S‐A mutants by QuikChange mutagenesis (Agilent). Generation of HEK293 cells expressing AP/V5 TMEFF2 and AP/V5 Δ_303‐320_TMEFF2 was described previously (Ali and Knäuper, 2007) and were maintained in DMEM with 10% FBS and 100 μg/mL hygromycin B at 37°C in a humidified incubator with 5% CO_2_. For shedding experiments 1 × 10^5^ HEK293 cells, expressing alkaline phosphatase tagged wild type or mutant TMEFF2, were plated per well into a 24 well plate and grown overnight in antibiotic free medium. 0.5 μg of expression plasmid encoding the active and inactive proteases in question were mixed with 1.5 μL of FuGENE 6 Transfection Reagent added to each well and grown for 2 days prior to shedding experiments in the presence or absence of ADAM 10, ADAM17 or general metalloproteinase inhibitor GM6001 described previously (Ali and Knäuper, 2007). Additional experiments were performed using DU145 cells transiently transfected with AP‐TMEFF2. Here, cell lysates were analysed for total AP‐activity to normalise the release of soluble AP‐TMEFF2 ectodomain into the medium following cell crowding experiments. The data are displayed as percentage of shed TMEFF2.

Cell lysates were harvested at the end of shedding experiments and analysed using 10% SDS–PAGE followed by Western blotting using PVDF membranes.

### Statistical analysis

Mean values ± SD from three independent experiments with four internal replicates were analysed using GraphPad Prism 5.0 and one‐way ANOVA with Tukey's test (**P < 0.01; *P < 0.05). *P*‐values below 0.05 were considered significant.

## Results and discussion

**Figure 1:**
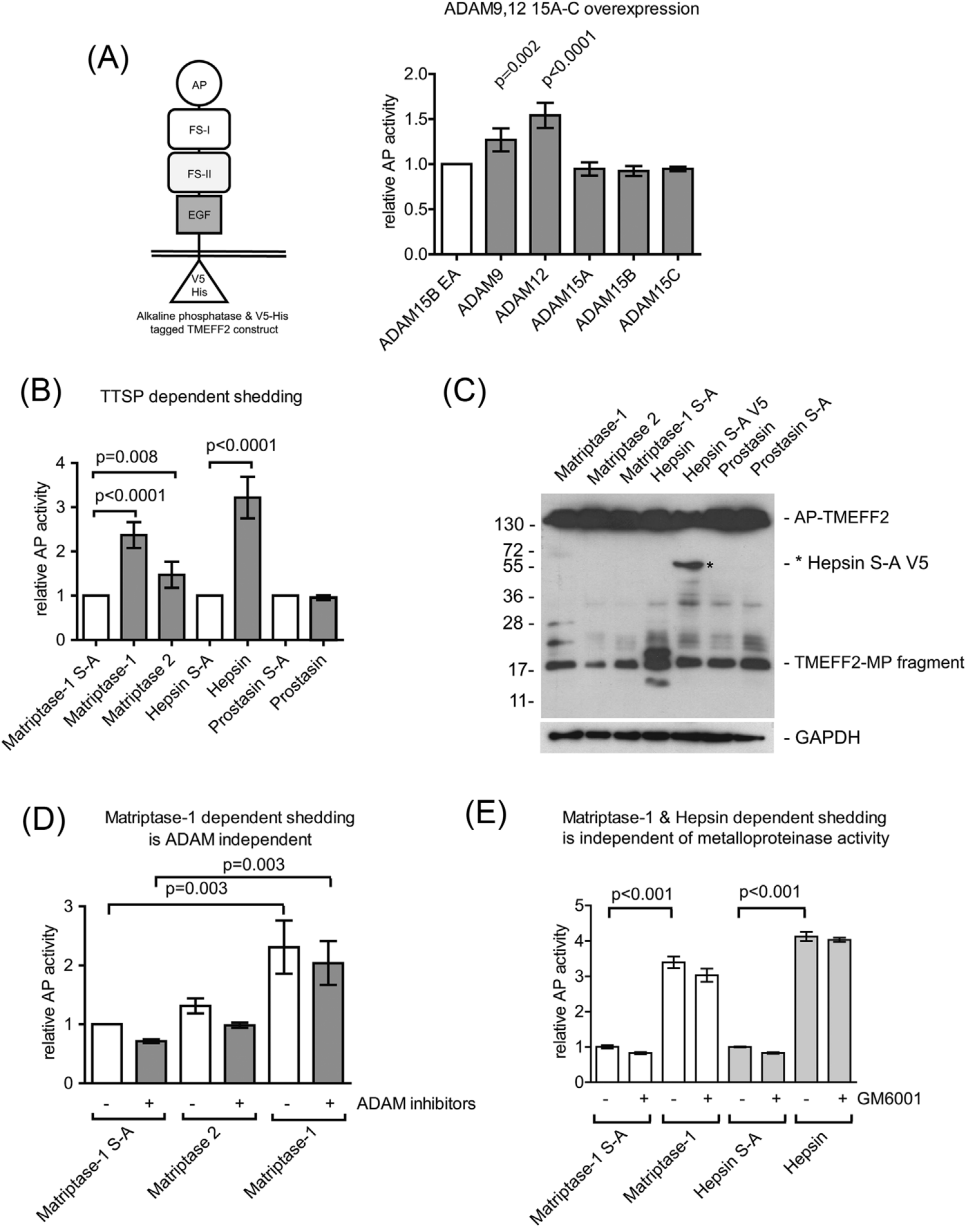
**TMEFF2 is a novel substrate for ADAM9, ADAM12, and type II transmembrane serine proteases (TTSPs)—matriptase‐1 and hepsin.** (A) Schematic representation of AP‐tagged TMEFF2 expression construct and ADAM9 and ADAM12‐dependent AP‐TMEFF2‐ECD release into media. (B) Release of AP‐TMEFF2‐ECD from cells transfected with matriptase‐1, matriptase‐2, hepsin, prostasin, or their inactive S‐A mutants. (C) WB analysis for the C‐terminal V5‐epitope of cell lysates demonstrating generation of distinct novel C‐terminal TMEFF2 fragments in matriptase‐1 (∼25 and 28 kDa) and hepsin (∼20 kDa) overexpressing cells. (D) The matriptase‐dependent release of TMEFF2‐ECD is independent of ADAM activity. (E) Matriptase‐1 and hepsin‐dependent TMEFF2 release is independent of metalloproteinase activity. MP fragment = metalloproteinase fragment.

Our first aim was to identify whether TMEFF2 could be targeted by other proteases accounting for soluble TMEFF2 (Quayle and Sadar, 2006). We assessed a panel of ADAMs with ADAM9 and ADAM12 overexpression leading to increased shedding of TMEFF2, while the expression of ADAM15 isoforms did not increase AP‐activity in media when compared to coexpression of an inactive ADAM15 EA mutant used as a transfection control (Figure 1A). C‐terminal TMEFF2 fragments were indistinguishable from previously described ADAM17 fragments (Ali and Knäuper, 2007) (not shown).

We then hypothesised that TTSPs could cleave TMEFF2, as matriptase‐1 is known to interact with TMEFF1 (Ge et al., 2006). Overexpression of matriptase‐1 or hepsin increased AP‐TMEFF2 fragment release into medium by 2–3.5‐fold, compared to inactive matriptase‐1/hepsin S‐A mutant coexpression. Matriptase‐2 overexpression was less efficient and increased AP‐activity in media 1.5‐fold, while prostasin had no effect (Figure 1B). Western blot analysis for remaining membrane‐associated fragments following TTSP cleavage showed novel, C‐terminal TMEFF2 fragments in matriptase‐1 (∼24 and ∼28 kDa) and hepsin (∼20 kDa) expressing cells (Figure 1C). These fragments were absent upon expression of their inactive S‐A mutant counterparts, in addition to the ∼17 kDa fragment which is due to background ADAM activity previously described (Ali and Knäuper, 2007). Therefore, matriptase‐1 and hepsin cleave TMEFF2 in different positions than ADAMs, generating novel transmembrane‐retained fragments. No additional C‐terminal fragments were detected in cells expressing matriptase‐2 (Figure 1C), despite increased AP‐activity levels in medium (Figure 1B). Thus, matriptase‐2 either cleaves TMEFF2 close to the ADAM cleavage site or alternatively activates ADAMs to induce proteolysis. To address this question, shedding experiments were performed in the presence of selective ADAM10 and ADAM17 inhibitors, GW280264X and GI254023X or the broad spectrum metalloproteinase inhibitor GM6001. The data in Figure 1D showed that matriptase‐2 dependent release of AP‐TMEFF2‐ECD required ADAM activity, whereas matriptase‐1‐dependent release did not. Additional experiments were performed to exclude contribution from ADAM9 and other metalloproteinases such as MMPs using the broad spectrum metalloproteinase inhibitor GM6001 (Maretzky et al., 2017) and cells overexpressing matriptase‐1 or hepsin, as well as their inactive counterparts. This analysis indicated that matriptase‐1 and hepsin were genuine TMEFF2 sheddases as inhibitor treatment was ineffective (Figure 1E).

**Figure 2:**
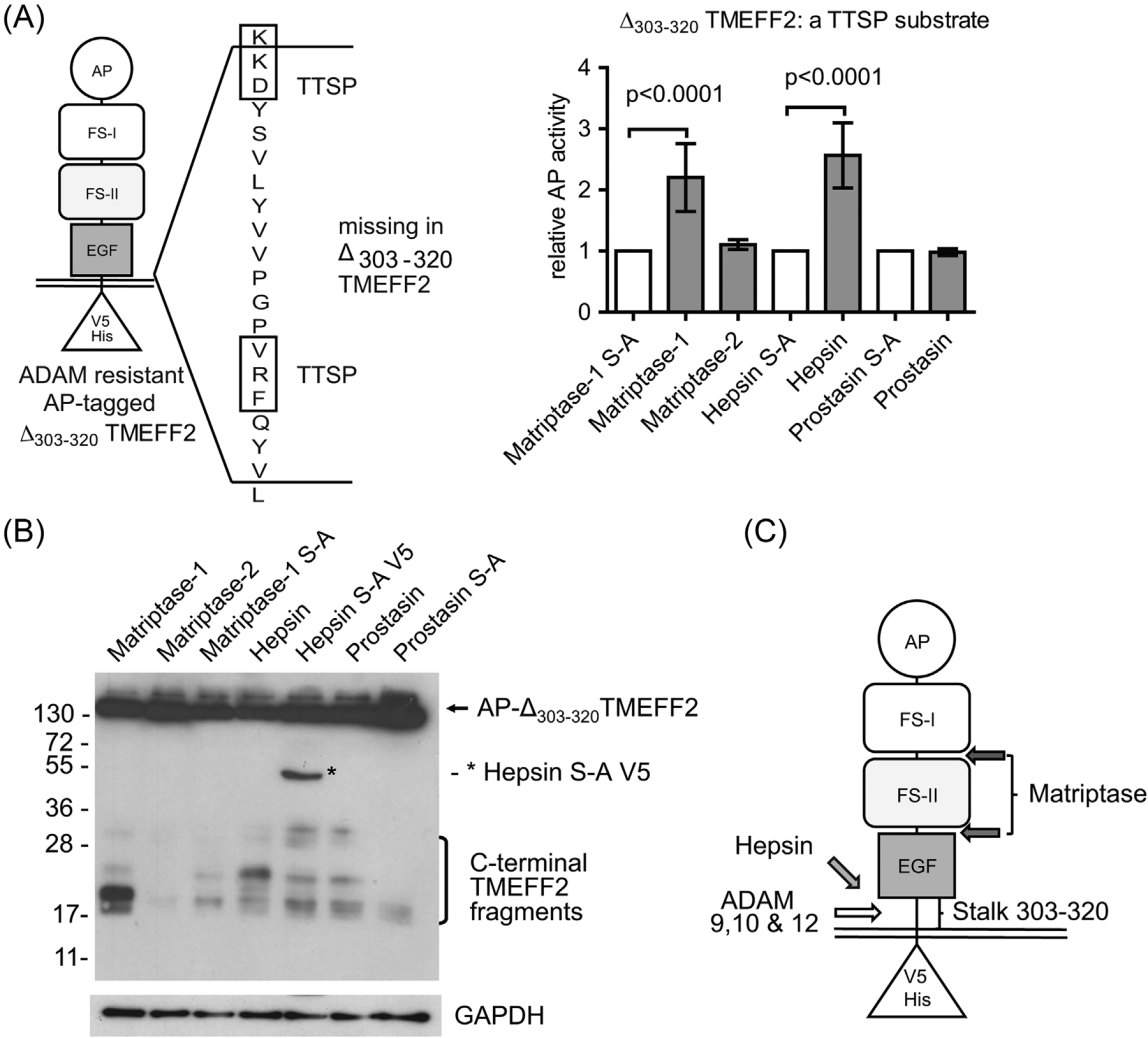
**Characterization of TTSPs cleavage sites using the AP‐Δ_303‐320_TMEFF2 mutant lacking the ADAM cleavage site.** (A) Schematic representation of AP‐Δ_303‐320_TMEFF2 expression construct and sequence motif deleted in this mutant, showing potential TTSP cleavage sites. Release of AP‐Δ_303‐320_TMEFF2 from cells co‐transfected with matriptase‐1, matriptase‐2, hepsin, prostasin, or their inactive S‐A mutants. (B) WB analysis of lysates for AP‐Δ_303‐320_TMEFF2 C‐terminal fragments shows distinct cleavage products for matriptase‐1 (∼17 & 23 kDa) and hepsin (∼25 kDa). (C) Model of predicted TMEFF2 cleavage sites for hepsin and matriptase‐1.

To corroborate these findings, ADAM‐cleavage‐resistant AP‐Δ_303‐320_ TMEFF2 (Ali and Knäuper, 2007) was used to confirm that matriptase‐1 and hepsin cleaved outside and matriptase‐2 within the stalk section containing the ADAM cleavage site. Both matriptase‐1 and hepsin cleaved AP‐Δ_303‐320_ TMEFF2 to a similar extent to wt TMEFF2 (Figure 2A), while matriptase‐2 was unable to directly cleave AP‐Δ_303‐320_TMEFF2 lacking the ADAM‐cleavage site. TMEFF2 C‐terminal fragment analysis in Figure 2B confirmed that matriptase‐1 and hepsin cleaved AP‐Δ_303‐320_ TMEFF2 by generating novel fragments, showing distinctly different molecular weights, when compared to cleaved wt AP‐TMEFF2. Matriptase‐1 produced 18 kDa and 20 kDa C‐terminal fragments, and hepsin a 25 kDa C‐terminal fragment of AP‐Δ_303‐320_TMEFF2. It has to be noted that the AP‐Δ_303‐320_TMEFF2 mutant also lacks two potential TTSPs cleavage site motifs, KKD and VRF (indicated in Figure 2A), as judged by the preferences of TTSPs for P1 arginine or P1′ lysine residues (Barré et al., 2014), although other sites can also be cleaved by TTSPs. Potentially, hepsin cleavage occurs at KKD in wild type TMEFF2 to produce the 20 kDa fragment and disruption of this site to the artificial sequence CEKLI then leads to an additional minor cleavage event, as seen when the AP‐Δ_303‐320_TMEFF2 mutant was cleaved in response to hepsin overexpression, producing a novel 25 kDa fragment. We predict possible cleavage sites for matriptase‐1 and hepsin in TMEFF2 (Figure 2C), with hepsin cleaving in the stalk sequence, releasing a soluble TMEFF2 fragment composed of the TMEFF2 ectodomain. Matriptase‐1 likely cleaves TMEFF2 in two positions, generating soluble proteins containing FS‐I or both FS modules, which in conjunction with ADAM dependent cleavage in the stalk region also liberates the EGF‐like domain. Thus, these cleavage events may be at least partially responsible for the generation of soluble TMEFF2 forms previously identified by others (Uchida et al., 1999; Quayle and Sadar, 2006).

Soluble TMEFF2 fragments containing FS‐domains generated by proteolysis likely modify PDGF‐AA growth factor signalling, where PDGF‐AA‐TMEFF2 complexes modify signalling through PDGFRα, thus full length TMEFF2 or soluble FS‐domain containing TMEFF2 fragments may block PDGF‐AA signalling (Lin et al., 2011). The FS domains of TMEFF2 also regulate corticotropine‐releasing hormone (CRH) signalling in corticotrope cells, where the production of cAMP, CREB and expression of pro‐opiomelanocortin was inhibited, resulting in decreased cell proliferation (Labeur et al., 2015). On the other hand, growth‐promoting activity of soluble TMEFF2 has been described (Horie et al., 2000; Ali and Knäuper, 2007; Chen et al., 2011a; Chen and Ruiz‐Echevarría, 2013), suggesting that this may be cell type dependent and potentially regulated by the pattern of ADAM and TTSP expression as well as by the growth factors present in the extracellular environment.

**Figure 3:**
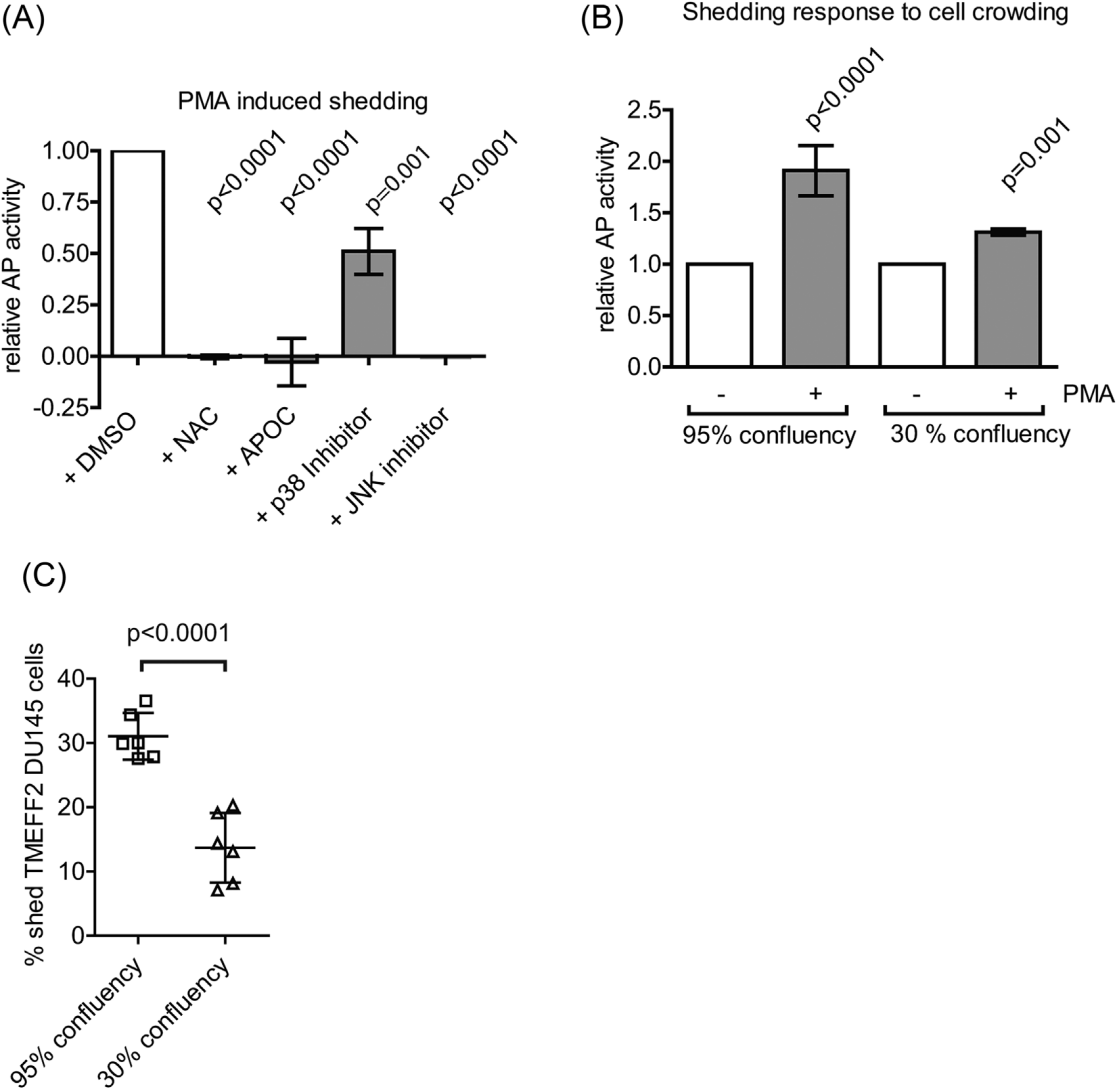
**Oxidative stress and ROS‐activated ADAMs participate in TMEFF2 shedding.** (A) PMA‐induced shedding from AP‐TMEFF2 HEK293 cells pre‐treated for 1 h with NAC, APOC, or p38 and JNK MAPKs inhibitors. (B) PMA‐induced shedding of AP‐TMEFF2 from 95% and 30% confluent cells. (C) TMEFF2 shedding in DU145 PCa cells is upregulated by cell crowding.

Reactive oxygen species (ROS) influence ADAM expression patterns and activation status (Sung et al., 2006; Willems et al., 2010) and they also regulate TTSP activity, as seen for matriptase‐1, which is activated by ROS (Chen et al., 2011b) thus adding additional layers of regulation of shedding events. ROS levels also regulate several signalling pathways in cancer (Hanahan and Weinberg, 2000; Liou and Storz, 2010), including PCa (Khandrika et al., 2009), which led us to hypothesise that ROS levels regulate TMEFF2 shedding. To investigate this hypothesis, we pre‐treated AP‐TMEFF2 HEK293 cells with the ROS scavenger NAC or the NADPH oxidase inhibitor APOC prior to stimulation with PMA, a known inducer of ROS generation (Datta et al., 2000) and ADAM17 activator (Brill et al., 2009). Both inhibitor treatments resulted in almost complete inhibition of AP‐TMEFF2‐ECD release, indicating ROS‐dependent TMEFF2 processing (Figure 3A). We hypothesised that ROS‐induced TMEFF2 shedding could be mediated by the stress‐activated protein kinases JNK or p38 to activate ADAM17. The JNK inhibitor completely blocked AP‐TMEFF2 release, whereas the p38 inhibitor reduced TMEFF2 shedding by 50% (Figure 3A) suggesting that TMEFF2 shedding is triggered by oxidative stress signalling. The growth of cells at high cellular density has been reported to be a contributing factor to the increased oxidative stress in cancer (Hanahan and Weinberg, 2000). We investigated whether shedding of TMEFF2 was a pathophysiological response triggered by oxidative stress originating from cells growing in high confluency conditions. Equal number of cells were plated onto 24‐well or 6‐well plates to obtain 95% and 30% confluency, respectively, and treated with equal volumes of medium containing PMA or control solvent. High cellular density significantly increased the shedding response to PMA activation (Figure 3B). To confirm that TMEFF2 shedding in response to cell crowding was also relevant in PCa cancer cells, we then transiently transfected DU145 cells with AP‐TMEFF2 cDNA and analysed shedding responses. AP‐activity in cell lysates was determined and AP‐activity in medium calculated as % shed TMEFF2 in cells grown at 3%0 or 95% confluency, respectively (Figure 3C). Data show significant increase in shedding in response to cell crowding, thus confirming the results obtained using HEK293 cells. Collectively, this suggests that cell crowding mimicking conditions frequently found in the tumour microenvironment may trigger TMEFF2‐ECD release, and contribute to the high proliferation rate of cancer cells.

In summary, differential TMEFF2 proteolysis producing various soluble fragments may be a general mechanism of changing its biological activity, dependent on the repertoire of ADAMs or TTSP expressed in the target cell, which in this case is regulated by ROS signalling.

## References

[cbin10832-bib-0001] Afar DEH , Bhaskar V , Ibsen E , Breinberg D , Henshall SM , Kench JG , Drobnjak M , Powers R , Wong M , Evangelista F , O'Hara C , Powers D , DuBridge RB , Caras I , Winter R , Anderson T , Solvason N , Stricker PD , Cordon‐Cardo C , Scher HI , Grygiel JJ , Sutherland RL , Murray R , Ramakrishnan V , Law DA (2004) Preclinical validation of anti‐TMEFF2‐auristatin E‐conjugated antibodies in the treatment of prostate cancer. Mol Cancer Ther 3(8): 921–32. 15299075

[cbin10832-bib-0002] Ali N , Knäuper V (2007) Phorbol ester‐induced shedding of the prostate cancer marker transmembrane protein with epidermal growth factor and two follistatin motifs 2 is mediated by the disintegrin and metalloproteinase‐17. J Biol Chem 282(52): 37378–88. 17942404 10.1074/jbc.M702170200

[cbin10832-bib-0003] Barré O , Dufour A , Eckhard U , Kappelhoff R , Béliveau F , Leduc R , Overall CM (2014) Cleavage specificity analysis of six type II transmembrane serine proteases (TTSPs) using PICS with proteome‐derived peptide libraries. PLoS ONE 9(9):e105984. 25211023 10.1371/journal.pone.0105984 PMC4161349

[cbin10832-bib-0004] Brill A , Chauhan AK , Canault M , Walsh MT , Bergmeier W , Wagner DD (2009) Oxidative stress activates ADAM17/TACE and induces its target receptor shedding in platelets in a p38‐dependent fashion. Cardiovasc Res 84: 137–44. 19482949 10.1093/cvr/cvp176 PMC2741344

[cbin10832-bib-0005] Brose N , Rosenmund C (2002) Move over protein kinase C, you've got company: alternative cellular effectors of diacylglycerol and phorbol esters. J Cell Sci 115(Pt 23): 4399–411. 12414987 10.1242/jcs.00122

[cbin10832-bib-0006] Chen X , Corbin JM , Tipton GJ , Yang LV , Asch AS , Ruiz‐Echevarría MJ (2014) The TMEFF2 tumor suppressor modulates integrin expression, RhoA activation and migration of prostate cancer cells. Biochim Biophys Acta 1843(6): 1216–24. 24632071 10.1016/j.bbamcr.2014.03.005 PMC4021708

[cbin10832-bib-0007] Chen X , Overcash R , Green T , Hoffman D , Asch AS , Ruiz‐Echevarría MJ (2011a) The tumor suppressor activity of the transmembrane protein with epidermal growth factor and two follistatin motifs 2 (TMEFF2) correlates with its ability to modulate sarcosine levels. J Biol Chem 286(18): 16091–100. 21393249 10.1074/jbc.M110.193805 PMC3091218

[cbin10832-bib-0008] Chen X , Ruiz‐Echevarría MJ (2013) TMEFF2 modulates the AKT and ERK signaling pathways. Int J Biochem Mol Biol 4(2): 83–94. 23936739 PMC3729255

[cbin10832-bib-0009] Chen C‐J , Wu B‐Y , Tsao P‐I , Chen C‐Y , Wu M‐H , Chan YLE , Lee H‐S , Johnson MD , Eckert RL , Chen Y‐W , Chou F , Wang J‐K , Lin C‐Y (2011b) Increased matriptase zymogen activation in inflammatory skin disorders. Am J Physiol Cell Physiol 300(3): C406–15. 21123732 10.1152/ajpcell.00403.2010 PMC3063967

[cbin10832-bib-0010] Chen L‐M , Zhang X , Chai KX (2004) Regulation of prostasin expression and function in the prostate. Prostate 59(1): 1–12. 14991861 10.1002/pros.10346

[cbin10832-bib-0011] Datta R , Yoshinaga K , Kaneki M , Pandey P , Kufe D (2000) Phorbol ester‐induced generation of reactive oxygen species is protein kinase cbeta‐dependent and required for SAPK activation. J Biol Chem 275(52): 41000–3. 11042219 10.1074/jbc.M009322200

[cbin10832-bib-0012] Dhanasekaran SM , Barrette TR , Ghosh D , Shah R , Varambally S , Kurachi K , Pienta KJ , Rubin MA , Chinnaiyan AM (2001) Delineation of prognostic biomarkers in prostate cancer. Nature 412(6849): 822–6. 11518967 10.1038/35090585

[cbin10832-bib-0013] Folgueras AR , de Lara FM , Pendás AM , Garabaya C , Rodríguez F , Astudillo A , Bernal T , Cabanillas R , López‐Otín C , Velasco G (2008) Membrane‐bound serine protease matriptase‐2 (Tmprss6) is an essential regulator of iron homeostasis. Blood 112(6): 2539–45. 18523150 10.1182/blood-2008-04-149773

[cbin10832-bib-0014] Fritzsche FR , Jung M , Tölle A , Wild P , Hartmann A , Wassermann K , Rabien A , Lein M , Dietel M , Pilarsky C , Calvano D , Grützmann R , Jung K , Kristiansen G (2008) ADAM9 expression is a significant and independent prognostic marker of PSA relapse in prostate cancer. Eur Urol 54(5): 1097–106. 18061337 10.1016/j.eururo.2007.11.034

[cbin10832-bib-0015] Ge W , Hu H , Ding K , Sun L , Zheng S (2006) Protein interaction analysis of ST14 domains and their point and deletion mutants. J Biol Chem 281(11): 7406–12. 16407223 10.1074/jbc.M510687200

[cbin10832-bib-0016] Gery S , Sawyers CL , Agus DB , Said JW , Koeffler HP (2002) TMEFF2 is an androgen‐regulated gene exhibiting antiproliferative effects in prostate cancer cells. Oncogene 21(31): 4739–46. 12101412 10.1038/sj.onc.1205142

[cbin10832-bib-0017] Glynne‐Jones E , Harper ME , Seery LT , James R , Anglin I , Morgan HE , Taylor KM , Gee JM , Nicholson RI (2001) TENB2, a proteoglycan identified in prostate cancer that is associated with disease progression and androgen independence. Int J Cancer 94(2): 178–84. 11668495 10.1002/ijc.1450

[cbin10832-bib-0018] Goel MM , Agrawal D , Natu SM , Goel A (2011) Hepsin immunohistochemical expression in prostate cancer in relation to Gleason's grade and serum prostate specific antigen. Indian J Pathol Microbiol 54(3): 476–81. 21934206 10.4103/0377-4929.85078

[cbin10832-bib-0019] Gray K , Elghadban S , Thongyoo P , Owen KA , Szabo R , Bugge TH , Tate EW , Leatherbarrow RJ , Ellis V (2014) Potent and specific inhibition of the biological activity of the type‐II transmembrane serine protease matriptase by the cyclic microprotein MCoTI‐II. Thromb Haemost 112(2): 402–11. 24696092 10.1160/TH13-11-0895

[cbin10832-bib-0020] Green T , Chen X , Ryan S , Asch AS , Ruiz‐Echevarría MJ (2013) TMEFF2 and SARDH cooperate to modulate one‐carbon metabolism and invasion of prostate cancer cells. Prostate 73(14): 1561–75. 23824605 10.1002/pros.22706 PMC3878307

[cbin10832-bib-0021] Hanahan D , Weinberg RA (2000) The hallmarks of cancer. Cell 100(1): 57–70. 10647931 10.1016/s0092-8674(00)81683-9

[cbin10832-bib-0022] Horie M , Mitsumoto Y , Kyushiki H , Kanemoto N , Watanabe A , Taniguchi Y , Nishino N , Okamoto T , Kondo M , Mori T , Noguchi K , Nakamura Y , Takahashi E , Tanigami A (2000) Identification and characterization of TMEFF2, a novel survival factor for hippocampal and mesencephalic neurons. Genomics 67(2): 146–52. 10903839 10.1006/geno.2000.6228

[cbin10832-bib-0023] Khandrika L , Kumar B , Koul S , Maroni P , Koul HK (2009) Oxidative stress in prostate cancer. Cancer Lett 282(2): 125–36. 19185987 10.1016/j.canlet.2008.12.011 PMC2789743

[cbin10832-bib-0024] Ko C‐J , Huang C‐C , Lin H‐Y , Juan C‐P , Lan S‐W , Shyu H‐Y , Wu S‐R , Hsiao P‐W , Huang H‐P , Shun C‐T , Lee M‐S (2015) Androgen‐induced TMPRSS2 activates matriptase and promotes extracellular matrix degradation, prostate cancer cell invasion, tumor growth, and metastasis. Cancer Res 75(14): 2949–60. 26018085 10.1158/0008-5472.CAN-14-3297

[cbin10832-bib-0025] Labeur M , Wölfel B , Stalla J , Stalla GK (2015) TMEFF2 is an endogenous inhibitor of the CRH signal transduction pathway. J Mol Endocrinol 54(1): 51–63. 25573902 10.1530/JME-14-0225

[cbin10832-bib-0026] Liang G , Robertson KD , Talmadge C , Sumegi J , Jones PA (2000) The gene for a novel transmembrane protein containing epidermal growth factor and follistatin domains is frequently hypermethylated in human tumor cells. Cancer Res 60(17): 4907–12. 10987305

[cbin10832-bib-0027] Lin K , Taylor JR , Wu TD , Gutierrez J , Elliott JM , Vernes J‐M , Koeppen H , Phillips HS , de Sauvage FJ , Meng YG (2011) TMEFF2 is a PDGF‐AA binding protein with methylation‐associated gene silencing in multiple cancer types including glioma. PLoS ONE 6(4): e18608. 21559523 10.1371/journal.pone.0018608 PMC3084709

[cbin10832-bib-0028] Lin H , Wada K , Yonezawa M , Shinoki K , Akamatsu T , Tsukui T , Sakamoto C (2003) Tomoregulin ectodomain shedding by proinflammatory cytokines. Life Sci 73(13): 1617–27. 12875894 10.1016/s0024-3205(03)00514-9

[cbin10832-bib-0029] Liou G‐Y , Storz P (2010) Reactive oxygen species in cancer. Free Radic Res 44: 479–96. 20370557 10.3109/10715761003667554 PMC3880197

[cbin10832-bib-0030] Lucas N , Day ML (2009) The role of the disintegrin metalloproteinase ADAM15 in prostate cancer progression. J Cell Biochem 106(6): 967–74. 19229865 10.1002/jcb.22087

[cbin10832-bib-0031] Maretzky T , Swendeman S , Mogollon E , Weskamp G , Sahin U , Reiss K , Blobel CP (2017) Characterization of the catalytic properties of the membrane‐anchored metalloproteinase ADAM9 in cell‐based assays. Biochem J 474(9): 1467–79. 28264989 10.1042/BCJ20170075 PMC8606101

[cbin10832-bib-0032] Maretzky T , Yang G , Ouerfelli O , Overall CM , Worpenberg S , Hassiepen U , Eder J , Blobel CP (2009) Characterization of the catalytic activity of the membrane‐anchored metalloproteinase ADAM15 in cell‐based assays. Biochem J 420(1): 105–13. 19207106 10.1042/BJ20082127

[cbin10832-bib-0033] Peduto L , Reuter VE , Sehara‐Fujisawa A , Shaffer DR , Scher HI , Blobel CP (2006) ADAM12 is highly expressed in carcinoma‐associated stroma and is required for mouse prostate tumor progression. Oncogene 25(39): 5462–6. 16607276 10.1038/sj.onc.1209536

[cbin10832-bib-0034] Peduto L , Reuter VE , Shaffer DR , Scher HI , Blobel CP (2005) Critical function for ADAM9 in mouse prostate cancer. Cancer Res 65(20): 9312–9. 16230393 10.1158/0008-5472.CAN-05-1063

[cbin10832-bib-0035] Quayle SN , Sadar MD (2006) A truncated isoform of TMEFF2 encodes a secreted protein in prostate cancer cells. Genomics 87(5): 633–7. 16439095 10.1016/j.ygeno.2005.12.004

[cbin10832-bib-0036] Riddick ACP , Shukla CJ , Pennington CJ , Bass R , Nuttall RK , Hogan A , Sethia KK , Ellis V , Collins AT , Maitland NJ , Ball RY , Edwards DR (2005) Identification of degradome components associated with prostate cancer progression by expression analysis of human prostatic tissues. Br J Cancer 92(12): 2171–80. 15928670 10.1038/sj.bjc.6602630 PMC2361819

[cbin10832-bib-0037] Sanders AJ , Parr C , Davies G , Martin TA , Lane J , Mason MD , Jiang WG (2006) Genetic reduction of matriptase‐1 expression is associated with a reduction in the aggressive phenotype of prostate cancer cells in vitro and in vivo. J Exp Ther Oncol 6(1): 39–48. 17228523

[cbin10832-bib-0038] Sanders AJ , Webb SL , Parr C , Mason MD , Jiang WG (2010) The type II transmembrane serine protease, matriptase‐2: possible links to cancer? Anticancer Agents Med Chem 10(1): 64–9. 20015002 10.2174/1871520611009010064

[cbin10832-bib-0039] Sreekumar A , Poisson LM , Rajendiran TM , Khan AP , Cao Q , Yu J , Laxman B , Mehra R , Lonigro RJ , Li Y , Nyati MK , Ahsan A , Kalyana‐Sundaram S , Han B , Cao X , Byun J , Omenn GS , Ghosh D , Pennathur S , Alexander DC , Berger A , Shuster JR , Wei JT , Varambally S , Beecher C , Chinnaiyan AM (2009) Metabolomic profiles delineate potential role for sarcosine in prostate cancer progression. Nature 457(7231): 910–4. 19212411 10.1038/nature07762 PMC2724746

[cbin10832-bib-0040] Sung S‐Y , Kubo H , Shigemura K , Arnold RS , Logani S , Wang R , Konaka H , Nakagawa M , Mousses S , Amin M , Anderson C , Johnstone P , Petros JA , Marshall FF , Zhau HE , Chung LWK (2006) Oxidative stress induces ADAM9 protein expression in human prostate cancer cells. Cancer Res 66(19): 9519–26. 17018608 10.1158/0008-5472.CAN-05-4375

[cbin10832-bib-0041] Takahashi S , Suzuki S , Inaguma S , Ikeda Y , Cho Y‐M , Hayashi N , Inoue T , Sugimura Y , Nishiyama N , Fujita T , Chao J , Ushijima T , Shirai T (2003) Down‐regulated expression of prostasin in high‐grade or hormone‐refractory human prostate cancers. Prostate 54(3): 187–93. 12518323 10.1002/pros.10178

[cbin10832-bib-0042] Uchida T , Wada K , Akamatsu T , Yonezawa M , Noguchi H , Mizoguchi A , Kasuga M , Sakamoto C (1999) A novel epidermal growth factor‐like molecule containing two follistatin modules stimulates tyrosine phosphorylation of erbB‐4 in MKN28 gastric cancer cells. Biochem Biophys Res Commun 266(2): 593–602. 10600548 10.1006/bbrc.1999.1873

[cbin10832-bib-0043] Webb SL , Sanders AJ , Mason MD , Jiang WG (2011) Type II transmembrane serine protease (TTSP) deregulation in cancer. Front Biosci (Landmark Ed) 16: 539–52. 21196187 10.2741/3704

[cbin10832-bib-0044] Willems SH , Tape CJ , Stanley PL , Taylor NA , Mills IG , Neal DE , McCafferty J , Murphy G (2010) Thiol isomerases negatively regulate the cellular shedding activity of ADAM17. Biochem J 428(3): 439–50. 20345372 10.1042/BJ20100179

[cbin10832-bib-0045] Zhong JL , Poghosyan Z , Pennington CJ , Scott X , Handsley MM , Warn A , Gavrilovic J , Honert K , Krüger A , Span PN , Sweep FCGJ , Edwards DR (2008) Distinct functions of natural ADAM‐15 cytoplasmic domain variants in human mammary carcinoma. Mol Cancer Res 6(3): 383–94. 18296648 10.1158/1541-7786.MCR-07-2028

